# Advances in Biomimetics: Patents from Nature

**DOI:** 10.3390/biomimetics11050303

**Published:** 2026-04-27

**Authors:** Stanislav N. Gorb, Longjian Xue, Barbara Mazzolai, Phillip B. Messersmith

**Affiliations:** 1 Department of Functional Morphology and Biomechanics, Zoological Institute, Kiel University, 24118 Kiel, Germany; 2 School of Power and Mechanical Engineering, Wuhan University, South Donghu Road 8, Wuhan 430072, China; xuelongjian@whu.edu.cn; 3 Bioinspired Soft Robotics (BSR), Istituto Italiano di Tecnologia (IIT), Via Morego 30, 16163 Genova, Italy; barbara.mazzolai@iit.it; 4 Departments of Bioengineering and Materials Science and Engineering, University of California, Hearst Memorial Mining Building, Berkeley, CA 94720-1760, USA; philm@berkeley.edu

Biomimetics seeks to translate principles from living systems into innovative engineering solutions by drawing on the remarkable efficiency, adaptability, and multifunctionality found in nature. Biological materials, structures, and processes are inherently hierarchical, combining mechanisms across nano-, micro-, meso-, and macroscales to achieve optimized performance under complex environmental conditions. Understanding these multiscale interactions is essential for identifying fundamental design rules and transferring them into technical applications. This Special Issue highlights recent progress in key areas of biomimetics, including materials and structures, surfaces and interfaces, locomotion and control, and biomedical systems, sometimes integrating and bridging these domains.

The contributions collected in this issue reflect a strong interdisciplinary effort, combining theoretical, experimental, and computational approaches from biology, physics, materials science, and engineering. They not only deepen our understanding of natural systems—from adhesive mechanisms and fluid transport to locomotion strategies and structural design—but also demonstrate how these insights can be translated into functional technologies. Particular emphasis is placed on the identification of underlying biological principles and their application in the development of advanced materials, surfaces, and adaptive devices. By bringing together diverse perspectives and methodologies, this Special Issue fosters the transfer of knowledge between disciplines and supports the continued advancement of biomimetic research toward practical and scalable innovations.

Among the contributions to this Special Issue, the review by Guidi et al. [1] addresses the pressing challenge of valvular heart disease, which continues to represent a major global health burden. Current prosthetic heart valves, although widely used, still fail to replicate the adaptive, regenerative, and long-term functional properties of native valves. Tissue-engineered heart valves (TEHVs) have therefore emerged as a promising alternative, representing living replacements capable of growth, remodeling, and physiological integration. However, despite considerable progress, their clinical translation remains limited, highlighting the need for design strategies that move beyond simple material substitution toward the development of functionally mature constructs. Adopting a biomimetic perspective, the authors emphasize native heart valves as a model system characterized by hierarchical organization, anisotropic mechanical behavior, mechanoresponsive cellular activity, and tightly regulated remodeling processes. The review integrates current knowledge in valve biology and mechanobiology with advances in scaffold design, biofunctionalization, biomechanical conditioning, and emerging fabrication technologies. Particular attention is given to how biomimetic approaches can reproduce extracellular matrix architecture, regulate immune and thrombotic responses, and guide tissue maturation through controlled mechanical stimulation. The authors conclude that successful clinical translation will require a coordinated design strategy that combines structural anisotropy, immune modulation, and dynamic conditioning, thereby illustrating how biomimetics can provide a unifying framework for the development of next-generation regenerative medical devices.

Biological adhesion and anti-adhesion represent two complementary strategies that are central to many natural systems and increasingly relevant for biomimetic applications. On the one hand, numerous organisms, such as insects, spiders, and geckos, have evolved highly efficient attachment mechanisms, while, on the other hand, plants have developed strategies to prevent adhesion or even actively trap animals. The contribution by Becker et al. [2] in this Special Issue investigates the mechanical properties of adhesive pad fluids in insects, which play a crucial role in mediating attachment. Using atomic force microscopy-based nanoindentation, the authors reveal that these fluids exhibit distinct viscoelastic behaviors, ranging from nearly inviscid to highly viscous or even rigid droplet types. Such variability suggests a non-uniform composition and indicates that insects may deploy functionally different fluid states to adapt adhesion to changing conditions. These findings advance our understanding of dynamic, fluid-mediated adhesion and provide inspiration for the development of soft, tunable, and reversible adhesive systems in engineering.

In contrast, anti-adhesive strategies are exemplified by carnivorous plants, such as pitcher plants, which exploit surface properties and fluid characteristics to capture prey. The study in this issue by Gorb et al. [3] demonstrates how different components of the pitcher trap syndrome—including trap geometry, inner surface coatings, and fluid properties—contribute to trapping efficiency. Biomechanical experiments using biomimetic artificial pitchers show that surfactant-containing fluids with strong wetting properties are particularly effective in immobilizing insects. In addition, microstructured coatings mimicking plant particular epicuticular waxes reduce contact between insect feet and surfaces and promote contamination of their adhesive organs, thereby impairing attachment. Together, these studies highlight the interplay between adhesion and anti-adhesion in biological systems and illustrate how both principles can be translated into innovative technologies, ranging from advanced gripping devices to anti-fouling and trapping surfaces.

The contribution by Varnier et al. [4] addresses fluid transport in biological and bioinspired systems, highlighting how directional control of flow emerges from structural design rather than active pumping alone. The authors investigate hydraulic asymmetries in tubular channels as a fundamental mechanism underlying biological valves, physiological transport systems, and engineered passive devices, such as Tesla valves. Using the concept of diodicity—defined as the ratio of pressure drops required to drive identical flow rates in opposite directions—the study systematically analyzes how geometry and internal structures generate directional bias in fluid transport. Through numerical simulations in both two- and three-dimensional configurations, the authors demonstrate that channel geometry alone establishes a baseline level of hydraulic asymmetry. This effect is further modulated by the presence of internal obstacles, which may be rigid or deformable, enabling either continuously open channels or valve-like structures capable of complete flow blockage. The extension to three-dimensional systems reveals that increased dimensionality and structural adaptability significantly enhance diodicity, amplifying directional control. These findings provide a unifying framework for understanding passive flow regulation in biological systems and offer valuable design principles for the development of efficient, low-energy biomimetic valves and microfluidic transport devices.

The contribution by Yang et al. [5] explores bioinspired robotic locomotion in aquatic environments, focusing on the integration of surface structure and interfacial fluid phenomena for controlled propulsion. The authors investigate shark skin as a multifunctional interface, combining a hierarchical arrangement of three-dimensional dermal denticles with an underlying structural layer that is known to contribute to passive drag reduction. Beyond this well-established function, the study addresses the less explored possibility of active drag modulation through surface-mediated fluid effects. To this end, the authors develop a 3D-printed swimming robot equipped with a porous substrate and biomimetic denticle structures, enabling a controlled release of surfactants that generate Marangoni-driven propulsion. By tuning denticle geometry and spatial arrangement, the system achieves programmable swimming trajectories and on-demand motion control. Additionally, the incorporation of superhydrophobic surface treatments reduces hydrodynamic resistance and further enhances propulsion efficiency. The experimental results show that denticle configurations with a 30° attack angle provide optimal performance under both hydrodynamic and aerodynamic conditions. These findings suggest that mucus secretion on shark skin may actively contribute to drag reduction via Marangoni effects, offering new principles for the design of autonomous, highly efficient aquatic robots.

This Special Issue also addresses bioinspired locomotion control in legged robotic systems, focusing on smooth and adaptive transitions between different gait patterns. Rostro-Gonzalez et al. [6] propose a control framework based on spiking neural networks functioning as central pattern generators (CPGs), which produce coordinated spike trains that emulate neural activity observed in biological systems. These signals are translated into synchronized motor commands, enabling a hexapod robot to execute distinct locomotion patterns, including walking, jogging, and running. A key aspect of the approach is the use of a similarity metric (SPIKE-synchronization) to identify optimal transition points between gaits, ensuring smooth switching without disrupting stability. This mechanism is complemented by force-sensing resistors embedded in the robot’s legs, which allow the real-time assessment of terrain stiffness and adaptive selection of the most appropriate locomotion pattern. Experimental validation on a physical hexapod robot across different surface conditions demonstrates improved balance, reduced abrupt actuator changes, and enhanced energy efficiency. Overall, the study [6] highlights how biologically inspired neural control strategies can significantly improve adaptability and robustness in legged robotic locomotion systems.

The authors included in this Special Issue also explore the use of biological neural network architecture for computational intelligence, demonstrating how the connectome of *Drosophila melanogaster* can be exploited as a reservoir for time-series prediction tasks. Costi et al. [7] utilize recently available full connectome data to construct an Echo State Network, directly mapping the biological connectivity matrix into a computational framework. By selecting subsets of highly connected neurons and applying different selection strategies that either preserve or modify the proportional representation of neuron classes, the authors derive computationally efficient reservoir architectures inspired by the fruit fly brain. The performance of these biologically derived reservoirs is evaluated against standard state-of-the-art implementations. The results show that connectome-based architectures exhibit significantly improved robustness to overfitting, particularly in regimes where conventional models tend to fail. Further analysis using hybrid configurations—combining biological topology with random synaptic weights and vice versa—demonstrates that both network structure and weight distribution contribute to the observed performance gains. Even when the full connectome is used as a reservoir, the system maintains strong generalization ability, achieving low prediction errors with reduced regularization. These findings highlight the potential of biological neural architectures as a powerful blueprint for developing more robust and efficient computational models in machine learning and artificial intelligence.

Another contribution in this Special Issue focuses on control strategies for bioinspired soft actuators in rehabilitation robotics, particularly pneumatic artificial muscles (PAMs), which are widely used due to their lightweight structure and inherent compliance. Brown and Xie [8] address the major challenge of accurately modeling and controlling the strongly nonlinear dynamics of PAM systems. To overcome these limitations, the authors develop a model predictive control (MPC) framework based on system models optimized using particle swarm optimization (PSO), enabling improved prediction and real-time motion control performance. The proposed MPC approach is systematically compared with classical PID control, PSO-tuned PID control, and iterative learning control (ILC) under a range of dynamic conditions, including varying setpoints, external loads, and disturbances. Experimental results show that conventional PID control exhibits the largest tracking errors and undesirable oscillations, while PSO-PID improves performance but still lacks stability in human–robot interaction scenarios. ILC achieves good convergence and disturbance rejection but struggles with varying input frequencies. In contrast, the MPC approach consistently demonstrates superior performance, achieving the lowest tracking errors, fast response to changing setpoints, and stable operation without requiring a learning phase. These results highlight the advantages of predictive, model-based control strategies for enhancing the safety, precision, and adaptability of soft robotic systems in biomedical applications.

A further contribution in this Special Issue highlights advances in bioinspired optics and functional nanostructured surfaces through the study of naturally occurring corneal architectures in arthropods. Kryuchkov et al. [9] address the challenge of producing efficient, cost-effective, and biocompatible nanopatterned coatings, which are essential for applications in cell signaling, drug delivery, and optical surface engineering. As a biological model system, beetles (Coleoptera) are investigated due to their highly diverse and adaptive corneal nanostructures, which serve both optical and protective functions. Using atomic force microscopy, the authors identify dynamic dimpled nanocoatings capable of transforming into maze-like or nipple-like surface morphologies. These transitions are interpreted as a result of reversible, self-assembled processes driven by surface adhesion effects. A key molecular component of this system, cuticular protein 7 (CP7), is identified and shown to possess intrinsic self-assembly properties that enable controlled nanopattern formation in vitro. By replicating these mechanisms, the study demonstrates a route toward tunable and reversible bioinspired nanocoatings. These findings not only provide insight into the evolutionary optimization of optical nanostructures in insects but also open new perspectives for the development of adaptive, bioinspired optical materials and surface technologies.

The final contribution in this Special Issue addresses bioinspired protective systems, focusing on how natural armors achieve an effective balance between mechanical protection and flexibility. Pantano and Baiamonte [10] investigate scale-like structures inspired by the segmented armor of fish and armadillos, where geometry and hierarchical organization play a crucial role in mechanical performance. In contrast to conventional flat artificial scales, the proposed designs feature three-dimensional architectures with a vertical support anchored to the substrate and an inclined protective surface, enabling a more realistic replication of biological armor functionality. Two distinct configurations—overlapping and staggered scales inspired by elasmoid fish structures—are analyzed using finite element simulations to evaluate puncture resistance and deformability. The results demonstrate that subtle geometric modifications can lead to pronounced differences in mechanical behavior: one configuration maximizes resistance to penetration, while the other enhances flexibility and compliance. This trade-off reflects a key principle in biological protective systems, where performance is governed by structural tuning rather than material changes alone. The study not only deepens the understanding of natural armor mechanics but also provides practical design guidelines for advanced protective equipment, including lightweight body armor, gloves, and thermal protection systems.

This Special Issue of *Biomimetics*, enriched by high-quality theoretical, experimental, and review contributions from researchers in biology, physics, materials science, and engineering, will undoubtedly find a broad and diverse readership among scientists engaged in this rapidly expanding field. The collected works not only demonstrate the depth and diversity of current biomimetic research but also highlight its strong interdisciplinary nature and growing technological relevance. By bridging fundamental biological insights with engineering applications—from adhesion and anti-adhesion systems to fluid transport, locomotion, nanostructured optics, and protective materials—this issue underscores the transformative potential of biomimetics as a unifying framework for innovation. It is expected to serve as a valuable reference for researchers, engineers, and designers seeking to translate principles from nature into next-generation technologies.

## References

[1] Guidi L., Rosellini E., Riccio G., Cascone M.G. (2026). Advancements and Challenges in Tissue-Engineered Heart Valves: Integrating Biomechanics, Biomaterials, and Biomimetic Design for Functional Maturity. Biomimetics.

[2] Becker M., Kovalev A.E., Büscher T.H., Gorb S.N. (2026). Mechanical Characterization of Stick Insect Tarsal Attachment Fluid Using Atomic Force Microscopy (AFM). Biomimetics.

[3] Gorb E.V., Lange M., Jamke A., Gorb S.N. (2026). Effect of Different Characters of the Pitcher Trap Syndrome in *Nepenthes* on Insect Trapping Efficiency: A Biomimetic Approach. Biomimetics.

[4] Varnier F., Norouzikudiani R., Corsi G., Agostinelli D., Levin I., DeSimone A. (2026). Hydraulic Asymmetries for Biological and Bioinspired Valves in Tubular Channels: A Numerical Analysis. Biomimetics.

[5] Yang K., Wang C., Jiang L., Fang R., Dong Z. (2025). Bioinspired Swimming Robots with 3D Biomimetic Shark Denticle Structures for Controlled Marangoni Propulsion. Biomimetics.

[6] Rostro-Gonzalez H., Guerra-Hernandez E.I., Batres-Mendoza P., Garcia-Granada A.A., Cano-Lara M., Espinal A. (2025). Enhancing Legged Robot Locomotion Through Smooth Transitions Using Spiking Central Pattern Generators. Biomimetics.

[7] Costi L., Hadjiivanov A., Dold D., Hale Z.F., Izzo D. (2025). The *Drosophila* Connectome as a Computational Reservoir for Time-Series Prediction. Biomimetics.

[8] Brown D.F., Xie S.Q. (2025). Model Predictive Control with Optimal Modelling for Pneumatic Artificial Muscle in Rehabilitation Robotics: Confirmation of Validity through Preliminary Testing. Biomimetics.

[9] Kryuchkov M., Wang Z., Valnohova J., Savitsky V., Karamehmedović M., Jobin M., Katanaev V.L. (2025). Smart Bio-Nanocoatings with Simple Post-Synthesis Reversible Adjustment. Biomimetics.

[10] Pantano A., Baiamonte V. (2025). Mechanics of Bio-Inspired Protective Scales. Biomimetics.

